# CALENDAR

**DOI:** 10.1038/sj.bjc.6990503

**Published:** 1999-06


					30 November 1999
Pre-Symposium Course: State-of-the-Art on Early
Diagnosis of Gynecological Cancer
Barcelona, Spain
Further information from:
Institut Universitari Dexeus, Department of Obstetrics and
Gynecology, Pso. de la Bonanova 67, 08017 Barcelona, Spain.
Tel: + 34 3 227 47 00, Fax: + 34 3 418 78 32, E-mail:
cripon@iudexeus.uab.es
30 November 3 December 1999
International Symposium: Gynecological Oncology '99
Barcelona, Spain
Further information from:
Institut Universitari Dexeus, Department of Obstetrics and
Gynecology, Pso. de la Bonanova 67, 08017 Barcelona, Spain.
Tel: + 34 3 227 47 00, Fax: + 34 3 418 78 32, E-mail:
cripon@iudexeus.uab.es
1 December 1999
French-Italian-Portuguese-Spanish Meeting on
Colposcopy
Barcelona, Spain
Further information from:
Institut Universitari Dexeus, Department of Obstetrics and
Gynecology, Pso. de la Bonanova 67, 08017 Barcelona, Spain.
Tel: + 34 3 227 47 00, Fax: + 34 3 418 78 32, E-mail:
cripon@iudexeus.uab.es
Errata
Br J Cancer 79(5/6): 865-867
The role of genetic factors in predisposition to squa-
mous cell cancer of the head and neck
The correct author names and affiliations for this paper are as
follows:
S Jefferies1
, R Eeles1
, D Goldgar2
, R AOHern1
, JM Henk1
,
M Gore1
, MPT Collaborators: P Rhys-Evans1
, D Archer1
,
K Bishop1
, E Solomon4
, S Hodgson4
, M McGurk4
, M OOConnell4
,
J Hibbert4
, D Easton5
and W Foulkes3
1
Institute of Cancer Research and the Royal Marsden Hospital,
Downs Road, Sutton, Surrey SM2 5PT, UK; 2
IARC, Lyon,
France; 3
Division of Medical Genetics, Department of Medicine,
Montreal General Hospital, McGill University, Montreal, Canada;
4
Guys Hospital, St Thomas' Street, London, UK; 5
IPH,
Cambridge, UK
Br J Cancer 79(9/10):1609-1612
Inducible nitric oxide synthase (iNOS) expression may
predict distant metastasis in human melanoma
W Tschugguel, T Pustelnik, H Lass, M Mildner, W Weninger, C
Schneeberger, B Jansen, E Tschachler, T Waldhr, JC Huber and
H Pehamberger
H Pehamberger's affiliations should have been listed as both
2
Division of General Dermatology and 6
Ludwig Boltzmann
Institute for Clinical and Experimental Oncology, University of
Vienna, School of Medicine, Wahringer Gurtel 18-20, A-1090
Vienna, Austria
The Acknowledgements should read `We are grateful to Helga
Herre and Ladislaus Szabo for excellent technical assistance and
helpful methodical discussions. The study was supported in part
by Schering Wien GesmbH and the Ludwig Boltzmann Institute
for Clinical and Experimental Oncology, Vienna, Austria'.
CALENDAR
1302
British Journal of Cancer (1999) 80(8), 1302
(c) 1999 Cancer Research Campaign
Article no. bjoc.1999.0503

				

